# Sirtfood intake in relation to the 10-year risk of major adverse cardiovascular events: a population-based cohort study

**DOI:** 10.1186/s12986-024-00798-9

**Published:** 2024-05-10

**Authors:** Mahdieh Golzarand, Saghar Estaki, Parvin Mirmiran, Fereidoun Azizi

**Affiliations:** 1 Nutrition and Endocrine Research Center, Research Institute for Endocrine Sciences, Shahid Beheshti University of Medical Sciences, No. 24, Shahid Arabi St., Yemen St., Chamran Exp., P. O. Box 193954763, Tehran, Iran; 2 Department of Clinical Nutrition and Dietetics, Faculty of Nutrition Sciences and Food Technology, National Nutrition and Food Technology Research Institute, Shahid Beheshti University of Medical Sciences, No. 7, Shahid Hafezi St., Farahzadi Blvd., Shahrak-e-qods, Tehran, 1981619573 Iran; 3 Endocrine Research Center, Research Institute for Endocrine Sciences, Shahid Beheshti University of Medical Sciences, Tehran, Iran

**Keywords:** Sirtuins, Cardiovascular, Food, Cohort study, SIRT

## Abstract

**Background:**

Sirtuins have an important role in the regulation of metabolic and biological processess. Thus, we hypothesized that foods that could activate sirtuins, known as “sirtfood”, may improve health status. So, this study was aimed at investigating the association between the amount of sirtfood intake and the risk of major adverse cardiovascular events (MACE).

**Methods:**

In this cohort study, 2918 adults who had no history of MACE at the start of the study (2006–2008) participated and were followed up on until 2018. The amount of sirtfoods intake (servings per week) was computed using a validated food frequency questionnaire. Each patient’s medical records were evaluated to detect MACE. The Cox proportional hazards model was applied to assess the association between the amount of sirtfood intake and the risk of MACE.

**Results:**

The median duration of the study was 10.6 years. The hazard ratio (HR) for the risk of MACE was 0.70 for the second (95% CI: 0.50, 0.98) and 0.60 (95% CI: 0.42, 0.86) for the third tertile of sirtfoods intake compared with the first tertile. This association was nonlinear, and sirtfoods consumption of more than five servings per week did not result in a lower risk of MACE. In addition, there was a significant interaction between age (P-interaction < 0.001) and sirtfoods intake in relation to MACE occurrence. When assessing sirtfood components, compared with the lowest intake, the highest amount of soy (HR: 0.74, 95% CI: 0.56, 0.99) and parsley (HR: 0.62, 95% CI: 0.47, 0.83) intake was related to a lower risk of MACE.

**Conclusion:**

Our results indicated an inverse association between a higher amount of sirtfood intake and a lower risk of MACE incidents. This association was nonlinear, and having more than five servings of sirtfood per week did not reduce the risk of MACE any further.

## Introduction

In the past few decades, the incidence of non-communicable diseases (NCDs) has increased dramatically and has become a major concern as, they not only affect the health status of a community but also result in severe economic problems [1]. Cardiovascular disease (CVD) is an example of a NCD that is considered to be the leading cause of death globally. According to the evidence, CVD is responsible for 17.9 million deaths annually, or 32% of all deaths worldwide [2]. Various factors are involved in CVD development, such as following an unhealthy diet, tobacco use, an inactive lifestyle, and alcohol misuse [3]. Oxidative stress derived from chronic inflammation also contributes to the pathogenesis of CVD [4].

Sirtuins (SIRTs) are a class of histone deacetylases that depend on nicotine-adenine dinucleotide (+) (NAD^+^) to act. SIRTs control important metabolic and biological pathways, including cell metabolism, caloric restriction mimetics, cell survival, senescence, proliferation, apoptosis, and deoxyribonucleic acid (DNA) repair [5]. They have also been associated with some age-related diseases, such as cancer, neurodegenerative diseases, and metabolic disorders [6]. Additionally, evidence has supported the role of SIRTs on cardiovascular health achieving by improving oxidative stress, endothelial function, dyslipidemia, angiogenesis, metabolic hemostasis, and aging changes [7–9]. Thus, compounds that activate SIRTs are interested in preventing and/or treating metabolic, vascular, and inflammatory diseases [10, 11]. The most well-known class of SIRT-activators are polyphenols, which include resveratrol, fisetin, quercetin, piceatannol, anthocyanidin, and quinine [12].

A number of studies have confirmed the beneficial effects of polyphenols on the cardiovascular system [13–17]. Polyphenols are found in fruits, vegetables, and cereals; however, they do not act as a single compound. Studies indicated that ingredients in a food have synergistic or antagonistic effects together [18]. So, evaluating the potential health benefits of a whole food that activates SIRTs may lead to more efficient and realistic results than assessing a single compound. In 2013, Pallauf et al. [19] proposed the term “sirtfood” to describe foods that activate SIRTs. We hypothesized that the sirtfood could improve health status. Therefore, the objective of this study was to investigate the association between sirtfood and the risk of major adverse cardiac events (MACE) in adults.

## Materials and methods

The data used in this cohort research was obtained from the third wave of the Tehran Lipid and Glucose Study (TLGS), conducted between 2006 and 2008. The initial purpose of TLGS was to reduce and prevent the risk of NCDs. As a brief description, 15,005 people who were at least three years of age or older participated in the first wave of the TLGS in 1999–2001 and were followed up at three-year intervals. More detailed descriptions of the goals and methods of TLGS have been elaborated elsewhere [20]. In the third wave of the TLGS, 12,519 eligible subjects participated. As the dietary assessment with a large sample size was costly and time-consuming, 4920 subjects were randomly selected based on age and sex to complete the dietary questionnaire. A total of 3462 subjects completed the questionnaire, and we selected 3055 of those who were older than 18 years for the current study. There were no differences in general characteristics between the selected subjects and the eligible subjects [21]. We excluded participants who had unclear or no data regarding their MACE status or previous history of MACE at baseline (*n* = 69) or missed follow-ups (*n* = 8). In addition, subjects who consumed below 1% or over 99% of the total energy intake (*n* = 58), meaning less than 916 kcal/d and more than 4990 kcal/d in women and less than 1026 kcal/d and more than 5890 kcal/d in men, were removed. In the end, 2918 participants were recruited and followed up until 2018.

All individuals received and filled out written consent forms in order to participate in the study. Also, the study protocol was approved by the ethical committee of Shahid Beheshti University of Medical Sciences.

### Measurements of demographic, anthropometric, and laboratory

Age, sex, occupation, education, smoking status, medications, and other demographic data were recorded using a questionnaire at baseline and during follow-up sessions by trained interviewers. Physical activity was assessed using the validated Persian version of the MAQ (Modifiable Activity Questionnaire) [22]. The level of physical activity was reported as metabolic equivalents in minutes per week (MET-min/week).

Participants’ body weight was measured barefoot and with the least amount of coverage on a digital scale (Seca, Humburg, Germany) with an accuracy of 100 g. Their height in a standing position was measured with a tape measure fixed on the wall with an accuracy of 0.5 cm. Then the body mass index (BMI) was calculated. Waist circumference (WC) was also obtained using a tape measure near the umbilicus without any pressure over light clothing. The measurement was recorded to the nearest 0.1 cm.

At each follow-up examination, blood samples were collected after 12–14 h of fasting. The enzymatic colorimetric method was used to determine triglyceride (TG) and total cholesterol (TC) concentrations by glycerol-3-phosphate oxidase and cholesterol oxidase in serum, respectively. High-density lipoprotein (HDL) was measured after precipitating lipoproteins containing Apo lipoprotein B with phosphotungstic acid. The low-density lipoprotein (LDL) level was calculated using the Friedwald formula [23]. Fasting serum glucose (FSG) was detected by a glucose oxidase assay. Blood sample analyses were performed by commercial kits (Pars Azmoun Company, Iran) and the Selectra auto-analyzer (Vital Scientific, Netherlands). At baseline and every three years, an expert physician monitored subjects’ systolic blood pressure (SBP) and diastolic blood pressure (DBP). Blood pressure was measured twice in the right arms using a mercury sphygmomanometer (Riester, Jungingen, Germany). The interval between measurements was 30 s, and the mean of the two values is considered blood pressure.

### Assessment of dietary intake and sirtfood

Dietary intake was assessed with a validated food frequency questionnaire (FFQ) [24] by experienced nutritionists every three years. The FFQ covered the daily, weekly, monthly, and yearly intake of 168 food items over the previous year. To estimate the daily intake of food items that were reported weekly, monthly, or yearly, the portion size of each food item multiplied the frequency of its consumption and was divided by 7, 30, or 365, respectively. The energy and nutrients’ consumption were estimated using the US Department of Agriculture’s (USDA) Food Composition Table (FCT) because the Iranian FCT does not offer comprehensive data. Nevertheless, the Iranian FCT was used for those local items that were not listed in the USDA FCT.

Twenty kinds of foods were indicated as sirtfood, including turmeric, arugula (rocket), extra-virgin olive oil, bird’s eye chili, blueberries, soy, buckwheat, capers, cereals, onions, coffee, dark chocolate (85% cocoa), kale, lovage, walnuts, matcha green tea, strawberries, medjool dates, parsley, red chicory, and red wine [25]. We reported sirtfood intake as the number of servings per week. Accordingly, the daily intakes in grams of each sirtfood were converted to its serving size and multiplied by seven. The list of sirtfood according to our FFQ was as follows: cereals (*Cerealis*), coffee (*Coffea*), soy (*Glycine max*), date (*Phoenix dactylifera*), strawberry (*Fragaria × ananassa*), walnut (*Juglans*), parsley (*Petroselinum crispum*), and onion (*Allium cepa*). To assess the link between the sirtfood intake and the MACE incidents, we calculated the cumulative average of each sirtfood intake. This was achieved by averaging the intake of each sirtfood at baseline and during the follow-up until the first MACE occurred, the date of death, the last visit, or the end of the follow-up.

### Definition of major adverse cardiac events

Data about cardiovascular events, deaths, and causes of death among TLGS participants is updated every year by a qualified nurse. Afterwards, the patients’ medical records or death certificates were collected by a physician for further evaluation. In the end, the TLGS outcome committee, including an internist, endocrinologist, cardiologist, epidemiologist, and other specialists, if necessary, examined the data and judged them. The MACE components included myocardial infraction (MI), stroke (either ischemic or hemorrhagic stroke), coronary heart disease (CHD), heart failure, unstable angina, and cardiovascular death [26].

### Statistical analysis

We reported continuous variables as mean ± standard error (SE) and categorical variables as count (%). The general characteristics of the participants across the tertiles of the sirtfood were compared using an analysis of covariance (ANCOVA) after adjusting for age and sex for continuous variables and Chi-squared for categorical variables. Dietary intake of each sirtfood item in the tertiles of the total sirtfood intake was reported after adjusting for gender, age, and total energy intake using the ANCOVA test. The association between the amount of total and individual sirtfood intake and the risk of MACE was assessed using the Cox proportional hazards model. The hazard ratio (HR) and 95% confidence interval (CI) were reported across tertiles of the sirtfood and per 1-serving increase in the amount of sirtfood intake. Schoenfeld residuals were used to test the proportional hazards (PH) assumption. To investigate the link between sirtfood components and outcomes of interest, the median of each sirtfood was calculated, and HR (95% CI) was reported as high vs. low intake of each food item. The first model was adjusted for sex and age. The second model was further adjusted for baseline BMI, SBP, TC, FSG, smoking status, residual WC (obtained from the regression of WC on BMI), physical activity, total energy intake, and flavonoid intake. We selected confounders based on literature and the data-driven method. In the last method, variables that changed the ratio of the sirtfood intake to MACE risk by > 5% were included in the model. The P-trend was determined using the median of each tertile. The person-years for each participant were calculated from baseline to the first MACE reported, the date of death, the last visit, or the end of follow-up. We also assessed the interaction between sirtfood intake and sex (male vs. female), age (< 50 years vs. ≥ 50 years), and BMI (< 30 kg/m^2^ vs. ≥ 30 kg/m^2^) in relation to MACE. Data was analyzed using SPSS software (version 20.0; IBM Corporation, Armonk, NY, USA) and Stata software (version 12; StataCorp, College Station, Texas, USA).

## Results

In this study, 2918 adults (male = 44.8%) with no previous history of MACE were participated and followed up for a median of 10.6 years. The average age of participants was 39.3 years. At baseline, 12.5% of participants had hypertension, 6.5% had diabetes, 52.4% had dyslipidemia, and 24.5% had obesity. A total of 3.2% of participants were taken anti-diabetic medications predominantly metformin and glibenclamide, while 9.3% were on heart medications such as beta-blockers, aspirin, angiotensin-converting enzyme (ACE) inhibitors, calcium channel blockers, diuretics, and vasodilators. General characteristics of participants across tertiles of sirtfood are presented in Table 1. Accordingly, subjects in the highest tertile of sirtfood were more female (*P* < 0.001), less smoked (*P* = 0.03), and more active (*P* < 0.001) in comparison to those in the lowest tertile of sirtfood. There were no significant differences in other factors between the tertiles of sirtfood.

**Table 1:** General characteristics of participants at baseline across tertiles of sirtfood (serving/week)

Characteristics	Tertile 1 (< 5.32)	Tertile 2 (5.32–8.93)	Tertile 3 (> 8.93)	*P*-value
Age (year)	38.8 ± 0.45	39.0 ± 0.45	40.2 ± 0.45	0.06
Male (%)	499 (51.3)	421 (43.3)	386 (39.7)	< 0.0001
Body mass index (kg/m^2^)	27.0 ± 0.15	26.8 ± 0.15	27.2 ± 0.15	0.23
Waist circumference (cm)	89.6 ± 0.38	88.9 ± 0.37	90.1 ± 0.37	0.07
Fasting serum glucose (mg/dL)	90.9 ± 0.69	90.8 ± 0.69	92.7 ± 0.69	0.10
Total cholesterol (mg/dL)	185 ± 1.15	186 ± 1.15	187 ± 1.15	0.41
Triglyceride (mg/dL)	139 ± 2.75	144 ± 2.74	146 ± 2.75	0.15
High-density lipoprotein (mg/dL)	42.9 ± 0.31	42.7 ± 0.31	42.8 ± 0.31	0.86
Low-density lipoprotein (mg/dL)	115 ± 1.02	115 ± 1.01	116 ± 1.01	0.70
Systolic blood pressure (mmHg)	111 ± 0.46	112 ± 0.46	112 ± 0.46	0.50
Diastolic blood pressure (mmHg)	73.6 ± 0.33	73.3 ± 0.32	73.4 ± 0.33	0.78
Weight changes from baseline (kg)	2.99 ± 0.20	3.27 ± 0.20	3.20 ± 0.20	0.60
Smoking (%)	109 (11.3)	98 (10.1)	75 (7.8)	0.03
Physical activity (MET-min/week)	707 ± 43.8	866 ± 41.3	1032 ± 40.5	< 0.0001

Data presented as mean ± standard error for continuous and number (percent) for non-continuous variables

P-value was obtained by ANCOVA test after adjustment for sex, age and chi-squared, as appropriate

Table 2 shows the total serving size as well as the serving size of individual sirtfood items consumed per week. The weekly average of sirtfood intake was 8 servings. In our population, onions (32.2%) and parsley (26.6%) were the main contributors to sirtfood, followed by cereals (14.3%).

**Table 2:** Dietary intake of participants across tertiles of sirtfood (serving/week)

Dietary intake	Tertile 1 (< 5.32)	Tertile 2 (5.32–8.93)	Tertile 3 (> 8.93)	*P*-value
Total energy (kcal/day)	2041 ± 22.3	2376 ± 22.2	2789 ± 22.3	< 0.0001
Sirtfood (serving/week)	4.00 ± 0.10	7.09 ± 0.10	13.1 ± 0.11	< 0.0001
Cereals (serving/ week)	0.58 ± 0.06	1.02 ± 0.06	2.08 ± 0.06	< 0.0001
Dates (serving/ week)	0.42 ± 0.04	0.79 ± 0.03	1.27 ± 0.04	< 0.0001
Soy (serving/ week)	0.12 ± 0.01	0.20 ± 0.01	0.30 ± 0.02	< 0.0001
Strawberry (serving/ week)	0.10 ± 0.02	0.14 ± 0.02	0.24 ± 0.02	< 0.0001
Walnut (serving/ week)	0.26 ± 0.03	0.46 ± 0.02	0.69 ± 0.03	< 0.0001
Parsley (serving/ week)	1.11 ± 0.08	1.86 ± 0.08	3.60 ± 0.08	< 0.0001
Onion (serving/ week)	1.28 ± 0.07	2.28 ± 0.06	3.95 ± 0.07	< 0.0001
Coffee (serving/ week)	0.14 ± 0.05	0.35 ± 0.05	1.01 ± 0.05	< 0.0001

Data presented as mean ± standard error

P-value was obtained by ANCOVA test after adjustment for sex, age, and total energy intake

**Fig. 1:**
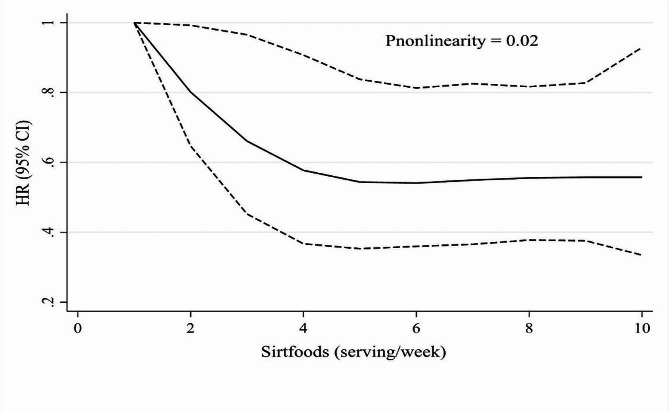
Nonlinear association between the amount of sirtfood intake (serving/week) and the risk of MACE incident

During the follow-up period, 203 subjects with established MACE were detected. Results of Cox regression analysis indicated no relationship between higher amount of sirtfood intake and the risk of MACE in the sex and age-adjusted model (Table 3, Model 1). However, after adjusting for all covariates, subjects in the second (5.32–8.93 servings/week) and third (> 8.93 servings/week) tertiles of sirtfood had a 30% (HR: 0.70, 95% CI: 0.50–0.98) and 40% (HR: 0.60, 95% CI: 0.42–0.86) lower risk of MACE compared with those in the first tertile (Table 3, Model 2). We found no association between 1-serving increases in the intake of sirtfood per week and the risk of MACE (Table 3, Model 2). The Schoenfeld residuals revealed no indication of PH assumption violation (*P* = 0.96). The restricted cubic spline revealed a nonlinear association between the sirtfood intake and the risk of MACE. The risk of MACE decreased along with increases in the amount of sirtfood consumption and reached a plateau above consuming 5 servings of sirtfood per week (Fig. 1, P-nonlinearity = 0.02).

**Table 3:** Hazard ratios (95% CI) for developing MACE across tertiles of sirtfood (serving/week)

Sirtfood intake	Tertile 1 (< 5.32)	Tertile 2 (5.32–8.93)	Tertile 3 (> 8.93)	*P*-trend	Per 1-serving/week	*P*-value
Person-years	9659	9953	9947	-	29,559	-
No. of cases	83/972	58/973	62/973	-	203/2918	-
Age and sex-adjusted model	1	0.73 (0.52, 1.02)	0.73 (0.53, 1.02)	0.07	0.99 (0.97, 1.02)	0.67
Multivariate-adjusted model	1	0.70 (0.50, 0.98)	0.60 (0.42, 0.86)	0.007	0.97 (0.95, 1.00)	0.07

The Cox proportional hazards models were used

Multivariate-adjusted model adjusted for sex, age, smoking, baseline BMI, smoking status, fasting serum glucose, total cholesterol, systolic blood pressure, residual waist circumference, physical activity, total energy intake, and flavonoid intake

There was a significant interaction between age (P-interaction < 0.001) and amount of sirtfood intake in relation to MACE occurrence, but not sex (P-interaction = 0.17) or BMI (P-interaction = 1.00). After categorizing the participants into groups based on their baseline age (< 50 years vs. ≥ 50 years), each serving per week increase in sirtfood was associated with a 4% lower risk of MACE in subjects 50 years of age and older (HR: 0.96, 95% CI: 0.93–0.99, *P* = 0.04). No relationship between sirtfood amount and the risk of MACE was detected in adults younger than 50 years old (Table 4).

**Table 4:** Hazard ratios (95% CI) for developing MACE across tertiles of sirtfood (serving/week) based on age categories

Dietary intake	Tertile 1	Tertile 2	Tertile 3	*P*-trend	Per 1-serving/week	*P*-value
**< 50 years old**						
Age and sex-adjusted model	1	0.58 (0.30, 1.14)	0.80 (0.43, 1.46)	0.52	1.01 (0.98, 1.05)	0.34
Multivariate-adjusted model	1	0.53 (0.27, 1.07)	0.52 (0.26, 1.04)	0.08	0.98 (0.96, 1.09)	0.37
**≥ 50 years old**						
Age and sex-adjusted model	1	0.79 (0.53, 1.16)	0.69 (0.46, 1.02)	0.07	0.98 (0.95, 1.01)	0.25
Multivariate-adjusted model	1	0.75 (0.50, 1.11)	0.60 (0.39, 0.91)	0.02	0.96 (0.93, 0.99)	0.04

The Cox proportional hazards models were used

Multivariate-adjusted model adjusted for sex, age, smoking, baseline BMI, smoking status, fasting serum glucose, total cholesterol, systolic blood pressure, residual waist circumference, physical activity, total energy intake, and flavonoid intake

The association between high vs. low adherence to individual sirtfood and the risk of MACE occurrence is presented in Table 5. Our findings indicated subjects with the highest intake of soy and parsley had a 26% (HR: 0.74, 95% CI: 0.56–0.99) and 38% (HR: 0.62, 95% CI: 0.47–0.83) lower risk of MACE compared with those with the lowest adherence to these foods, respectively. There was no relationship between other sirtfoods and MACE.

**Table 5:** Hazard ratios (95% CI) for developing MACE by high vs. low intake of individual sirtfood

Sirtfoods	Model 1	*P*-value	Model 2	*P*-value
Cereals	0.90 (0.69, 1.20)	0.50	0.89 (0.67, 1.18)	0.40
Dates	0.86 (0.65, 1.13)	0.29	0.84 (0.64, 1.12)	0.24
Soy	0.73 (0.55, 0.97)	0.03	0.74 (0.56, 0.99)	0.04
Strawberry	0.96 (0.73, 1.26)	0.77	0.89 (0.68, 1.18)	0.44
Walnut	0.94 (0.72, 1.27)	0.67	0.94 (0.72, 1.24)	0.67
Parsley	0.67 (0.50, 0.88)	0.005	0.62 (0.47, 0.83)	0.001
Onion	0.98 (0.75, 1.29)	0.89	0.85 (0.64, 1.12)	0.24
Coffee	0.96 (0.70, 1.31)	0.78	0.99 (0.73, 1.37)	0.98

The Cox proportional hazards models were used

Model 1 adjusted for sex and age

Model 2 adjusted for sex, age, smoking, baseline BMI, smoking status, fasting serum glucose, total cholesterol, systolic blood pressure, residual waist circumference, physical activity, total energy intake, and flavonoid intake

## Discussion

In the present study, a higher amount of sirtfood intake was inversely associated with the 10-year risk of MACE in a nonlinear manner. These results are crucial for protecting against the risk of MACE, especially in adults older than 50 years old. In addition to the amount of sirtfood, we found an inverse association between the highest intakes of soy and parsley and the risk of MACE incidents.

As far as we are aware, cohort studies examining the relationship between the amount of sirtfood consumption in the diet and the incidence of MACE are rare. In contrast, several cohort studies have assessed the cardiovascular protective effects of certain sirtfood individually. According to a meta-analysis by Zuo et al. [27], soy consumption was associated with a lower risk of CHD (RR: 0.84; 95% CI: 0.74–0.94, *I*^*2*^ = 64.5%, *n* = 12) and marginally with CVD (RR: 0.94; 95% CI: 0.88-1.00, *I*^*2*^ = 68.4%, *n* = 23). They reported no relationship between soy and the risk of stroke (RR: 0.96; 95% CI: 0.87–1.05, *I*^*2*^ = 73.2%, *n* = 13). Similar to soy, the protective effects of cereals, coffee, and walnuts against cardiovascular events were also reported by other meta-analyses. The documents showed that the relative risk (RR) for CVD was 0.78 (95% CI: 0.73–0.85, *I*^*2*^ = 40%, *n* = 10) for every three-servings/day increase in whole grain intake [28], 0.89 (95% CI: 0.84–0.94, *n* = 35) for every 1.5 cup/day coffee drink [29], and 0.81 (95% CI: 0.70–0.92, *I*^*2*^ = 73%, *n* = 3) for the highest category of walnut intake compared with the lowest category [30]. In the present study, in addition to the amount of sirtfood, we found an inverse relationship between high intakes of soy and parsley and the risk of MACE, but not with other sirtfoods. It is worth mentioning that the soy and parsley intake comprise about one-third of the sirtfood proportion of our participants’ diet. Therefore, we cannot relate the inverse association between sirtfood and MACE to the aforementioned sirtfoods. It is possible that the found association may be attributed to the synergistic interactions between various sirtfoods in the diet. In addition, the variability of sirtfood consumed by TLGS participants must be considered when interpreting our findings. Because there was no data about consuming some sirtfood in our FFQ, such as turmeric or bird’s eye chili, and some of them were not consumed routinely by our population, like arugula or red chicory, which may affect results.

In the current study, we hypothesized that sirtfood, by stimulating SIRTs, may be involved in the prevention of MACE. Evidence indicates that SIRTs, by suppressing pro-inflammatory cytokines and reducing oxidative stress, modulating endothelial function, enhancing the gut microbiota composition, and improving metabolic profiles, may potentially prevent cardiovascular events [19, 25]. In vivo and in vitro studies have revealed that the activation of SIRTs by polyphenols may ameliorate CVD [31–35]. However, we cannot extrapolate these findings to humans, even though relevant human studies are scarce to support our findings. In this study, we found that the inverse association between sirtfood and the risk of MACE was independent of dietary flavonoids. It is likely that the other ingredients included in sirtfood via stimulation SIRTs may be responsible for the observed relationship. Opstad et al. [36] in a prospective randomized control trial on healthy subjects aged > 69 years (*n* = 326) assessed the effect of selenium (200 µg/d) plus coenzyme Q10 (200 mg/d) supplementation on SIRT1 concentration and the 10-year risk of cardiovascular mortality. During four years of supplementation, SIRT1 levels increased significantly in the intervention group compared to the placebo (i.e., baker’s yeast and vegetable oil with added vitamin E) group. After 10 years of follow-up, a lower number of cardiovascular deaths was reported among the intervention group compared with the placebo group, which was associated with a higher level of SIRT1. Results of two separate trials also indicated that an increased SIRT1 level after calorie restriction or resveratrol supplementation was associated with an improvement in HDL composition, body fat mass, and the body’s total antioxidant capacity, all of which are risk factors for cardiovascular events [37, 38]. These results suggest that sirtfood might be an effective dietary plan for the prevention of cardiovascular events, thereby preserving health status. In the present study, we were unable to measure the SIRTs at baseline and follow-ups. So, we could not compare SIRTs between subjects who consumed more sirtfood and those who had the lowest intake. In future investigations, assessing the SIRTs is advised to infer whether a possible inverse relationship between the amount of sirtfood and MACE depends on the increase in the SIRTs.

This study has several strengths and limitations. The follow-up period was too lengthy to detect MACE. The long-term effect of sirtfood on the risk of MACE was assessed by calculating the cumulative average of sirtfood for each participant. Exposure and outcome were not self-reported. However, there was limited variability in the sirtfood consumed in our FFQ. We could not take into account all sirtfood in the present study as a result of a lack of access to or data in our FFQ on them. So, our findings may not generalize to other populations. Besides, we were unable to consider all MACE risk factors, such as alcohol consumption or socio-economic status, in the final model due to a paucity of data in our questionnaire.

## Conclusion

Our results indicated an inverse association between sirtfood intake and a lower risk of MACE incidents. This association had a nonlinear trend, as the risk of MACE decreased with an increasing amount of sirtfood when it was five or less, but it was not associated with a further reduction in risk if the amount was greater than five.

## Data Availability

No datasets were generated or analysed during the current study.

## Abbreviations

ANCOVA: Analysis of covariance
BMI: Body mass index
CHD: Coronary heart disease
CVD: Cardiovascular disease
DBP: Diastolic blood pressure
eNOS: Endothelial nitric oxide synthase
FCT: Food Composition Table
FSG: Fasting serum glucose
FFQ: Food frequency questionnaire
HDL: High-density lipoprotein
HR: Hazard ratio
iNOS: Inducible nitric oxide synthase
LDL: Low-density lipoprotein
MACE: Major adverse cardiac events
MAQ: Modifiable activity questionnaire
MET: Metabolic equivalent
NAD^+^: nicotine-adenine dinucleotide (+)
NCDs: Non-communicable diseases
NF-κB: Nuclear factor kappa B cells
SBP: Systolic blood pressure
SE: Standard error
SIRTs: Sirtuins
TC: Total cholesterol
TG: Triglycerides
TLGS: Tehran Lipid and Glucose Study
USDA: US Department of Agriculture
WC: Waist circumference
